# Acute hospital use in older adults following the 2015 Dutch reform of long-term care: an interrupted time series analysis

**DOI:** 10.1016/S2666-7568(23)00064-8

**Published:** 2023-06

**Authors:** Joost D Wammes, Pieter Bakx, Bram Wouterse, Bianca M Buurman, Terrence E Murphy, Janet L MacNeil Vroomen

**Affiliations:** Department of Internal Medicine, Section of Geriatric Medicine, Amsterdam Public Health Research Institute, Amsterdam UMC, University of Amsterdam, Amsterdam, Netherlands; Department of Social and Behavioral Sciences, Yale School of Public Health, New Haven, CT, USA; Erasmus School of Health Policy and Management, Erasmus University Rotterdam, Rotterdam, Netherlands; Erasmus School of Health Policy and Management, Erasmus University Rotterdam, Rotterdam, Netherlands; Department of Internal Medicine, Section of Geriatric Medicine, Amsterdam Public Health Research Institute, Amsterdam UMC, University of Amsterdam, Amsterdam, Netherlands; Department of Public Health Sciences, Pennsylvania State College of Medicine, Hershey, PA, USA; Department of Internal Medicine, Section of Geriatric Medicine, Amsterdam Public Health Research Institute, Amsterdam UMC, University of Amsterdam, Amsterdam, Netherlands

## Abstract

**Background:**

In 2015, the Dutch government implemented a long-term care (LTC) reform primarily designed to promote older adults to age-in-place. Increased proportions of older adults living in the community might have resulted in more and longer acute hospitalisations. The aims of this study were to evaluate whether the Dutch 2015 LTC reform was associated with immediate and longitudinal increases in the monthly rate of acute clinical hospitalisation and monthly average hospital length of stay (LOS) in adults aged 65 years or older.

**Methods:**

In this interrupted time series analysis of national hospital data (2009–18), we evaluated the association of the Dutch 2015 LTC reform with the monthly rate of acute clinical hospitalisation and monthly average LOS for older adults (aged ≥65 years). Patient-level episodic hospital data were provided by Dutch Hospital Data. Records were included that were defined as an acute clinical hospital admission for which a medical specialist decided treatment was necessary within 24 h. The analysis controlled for population growth (Dutch population data was provided by Statistics Netherlands) and seasonality, and calculated adjusted incident rate ratios (IRR).

**Findings:**

Before the 2015 LTC reform, the rate of acute monthly hospitalisation was increasing (IRR 1·002 [95% CI 1·001–1·002]). A positive average reform effect was observed (1·116 [1·070–1·165]), accompanied by a negative change in trend (0·997 [0·996–0·998]) that resulted in a decreasing trend over the post-reform period (0·998 [0·998–0·999]). The pre-reform trend of LOS was decreasing (0·998 [0·997–0·998]), and the 2015 reform exhibited a positive change in trend (1·002 [1·002–1·003]) that resulted in a stabilisation of LOS in the post-reform period (0·999 [0·999–1·000]).

**Interpretation:**

Our findings suggest that the increase in the rate of acute hospitalisation after the reform implementation was temporary, whereas the increase in LOS post-reform appeared to last longer than expected. These results have the potential to inform policy makers about effects of ageing-in-place LTC strategies on health and curative care.

**Funding:**

The Netherlands Organization for Health Research and Development, the Yale Claude Pepper Center, and the National Center for Advancing Translational Sciences, National Institutes of Health.

## Introduction

The Netherlands is the highest spender on long-term care (4·6% of its gross domestic product in 2020) among all Organisation for Economic Co-operation and Development countries.^1^ The Dutch long-term care (LTC) system provides a broad range of health and social services, wherein nearly all costs of home and institutional care are publicly financed with a low level of co-payments (8% in 2014). To control growing costs, a major reform of the Dutch LTC system was implemented on Jan 1, 2015.^2,3^ The LTC reform was centred around four main principles: (1) a shift from residential care to ageing-in-place; (2) a more social and community-based approach to LTC; (3) decentralisation of community-based care from the regional to the municipal level; and (4) fiscal sustainability of the LTC system.^2,3^

Since the reform implementation, the proportion of older adults (aged ≥65 years) who age-in-place has grown rapidly. This growth is reflected by the decrease in the total number of older adults residing in long-term care facilities (−5·2% from 2015 to 2017 compared with +0·3% from 2013 to 2015).^4^ Also, it is probable that the ageing-in-place population includes more older adults living with frailty. Since 2013, residential care for people with low care needs has been abolished; however, the Dutch 2015 LTC reform implemented even stronger measures, by restricting LTC facility admission to only those adults who require 24-h care or supervision per day. Older adults who previously would have been admitted to an LTC facility before the reform are now dependent on homecare services and informal care. Accompanying a greater reliance on home care, the responsibility of home care was decentralised to the municipalities, who expressed great difficulties with accommodating their new responsibilities.^3,5^ The budget that municipalities received for providing home care was 11% lower after the reform, whereas the budget for providing domiciliary care was 35% lower.^2^ Furthermore, before the reform home care was a legal entitlement. However, it became a provision, after the reform implementation, with more discretionary power for municipalities to decide on who would get care and how much.^6^ Still, the amount of people using home care increased by 8·8% from 2015 to 2017.^7^

The increased proportion of older adults living in the community with frailty, and changes in the provision of home care, might have resulted in more acute hospitalisations and extended hospital stays. Individuals who would otherwise have been treated within the LTC facility now have to visit the hospital or can have higher hospitalisation risks due to the negative health effects of living at home such as an increased risk of falls. This change in provision could impact person-centred outcomes such as quality of life, which is important to evaluate when assessing policy changes.^8^ Before the reform, the number of acute hospital visits among Dutch older adults was increasing,^9^ and length of stay (LOS) in the hospital was declining.^10^ MacNeil Vroomen and colleagues^11^ showed that the 2015 Dutch LTC reform was associated with increased risk of hospital death for older Dutch adults, suggesting an increase in hospital use due to the reform. However, it is unclear if acute hospitalisation rates and LOS of those hospitalisations have increased in the older population of Dutch adults since the introduction of the reform.

Acute hospital admissions are a driver of health-care expenditures and are associated with adverse health outcomes in older adults.^12^ Extended LOS can increase costs and result in extra disease burden for patients.^13^ Therefore, understanding the association between the Dutch 2015 LTC reform and hospital use is necessary to inform patients, providers, payers, and policy makers about how trends in ageing-in-place might be associated with trends in hospitalisations. The aims of this study were to evaluate whether the Dutch 2015 LTC reform was associated with immediate and longitudinal increases in the monthly rate of acute clinical hospitalisation and in the monthly average LOS among Dutch adults aged 65 years and older. All these large policy changes in the entire LTC system became effective immediately on Jan 1, 2015. Because of the sudden nature of this implementation, we hypothesise that the reform is associated with immediate increases in rate of hospitalisation and LOS. We also hypothesise that the reform is associated with longitudinal increases in rate of acute hospitalisation and LOS due to the larger proportion of frail older adults living in Dutch communities who would require complex social and medical care.

## Methods

### Study design

Interrupted time series analyses are commonly used to study associations between health policy interventions and population-level health outcomes. We used an interrupted time series analysis to evaluate whether the Dutch 2015 LTC reform was associated with an immediate (level) and longitudinal (trend) change in monthly acute clinical hospitalisation rate and change in monthly average LOS of those hospitalisations. Analysis was based on the guidelines of Bernal and colleagues^14^ and the STROBE guidelines^15^ were used to structure reporting and interpretation of results (appendix 2 pp 1–2).

### Data and study population

Dutch Hospital Data provided patient-level episodic hospital data for the years 2009–18 from all hospitals in the Netherlands, covering the total Dutch population aged 65 years and older. We included records that were defined as an acute clinical hospital admission for which a medical specialist decided treatment was necessary within 24 h.^16^ Statistics Netherlands provided records from the compulsory municipal resident register for the years 2009–18 covering the total Dutch population that were used to calculate a monthly population size of adults aged 65 years and older, and to identify patient characteristics such as age and sex. Hospital and municipality data were linked by an anonymised population register number with nearly perfect matching (>99%). We collapsed patient level data to aggregated population-level monthly time intervals. Data on race or ethnicity were not present in the dataset. Ethical approval was not required as data were anonymised and aggregated at the population level.

### Primary outcomes of hospital use

The outcome of monthly acute clinical hospitalisation rate was based on the total monthly count of hospitalisations and the monthly weighted population aged 65 years and older as an offset. The outcome for monthly average LOS was determined by averaging all LOS for hospitalisations of Dutch people of 65 years or older within a given month. For each person-hospitalisation, LOS was determined by subtracting the discharge date from the admission date. Acute clinical hospitalisations with a stay of less than 24 h were counted as 1 day.

Other variables included time in months (continuous) since the start of the study, a reform dummy (where pre-reform period is the reference), and their interaction. A seasonality variable was also constructed within each year by including monthly radians from sine and cosine functions as described by Doyle and colleagues.^17^

### Statistical analysis

Descriptive statistics were used to present patient characteristics, and to calculate yearly unadjusted rates of hospitalisation and monthly average LOS over the entire timeline of the study. The two outcomes were subsequently compared between the pre-reform and post-reform periods.

After evaluating which distribution was the best fit by minimisation of the Bayesian Information Criterion (BIC),^18^ a generalised linear model with a negative binomial distribution and the log link function was used to model monthly rates of acute hospitalisation and monthly average LOS. Acknowledging that the median is sometimes preferred for skewed data such as the LOS,^19^ the BIC provided strong evidence in favour of modelling the monthly mean (appendix 2 p 3). We regressed the outcomes of monthly rate of hospitalisation and monthly average LOS $\left(Y_{t}\right)$ on time in continuous months (T), an indicator of reform $\left(X_{1}\right)$, and their interaction $\left(TX_{1}\right)$, using the following interrupted time series model regression (model details are in appendix 2 [p 3]):

$$\log Y_{t}={\text{β}}_{0}+{\text{β}}_{1}T+{\text{β}}_{2}X_{t}+{\text{β}}_{3}TX_{t}$$

${\text{β}}_{0}$ represents the outcome at the start of data collection (January, 2009). Time can be interpreted as the pre-reform trend—ie, the monthly change in outcome in the pre-reform period $\left({\text{β}}_{1}\right)$. The reform indicator $\left({\text{β}}_{2}\right)$ can be interpreted as the average change in the outcomes associated with the reform (ie, average reform effect). The interaction term $\left({\text{β}}_{3}\right)$ can be interpreted as the change in trend between the pre-reform and post-reform trends. Additionally, post-reform trends (ie, the monthly change in outcome in the post-reform period) were calculated as the linear combinations of the estimated pre-reform trend (time) and trend change (interaction term). To account for the dependence between observations, models were adjusted for seasonality by including sine and cosine functions,^17^ in addition to applying robust confidence intervals. Because the model is fitted on the log of the outcome, all results are reported as incident rate ratios (IRR), which are the exponentiation of the regression coefficients and their corresponding 95% CIs. For example, an IRR greater than 1 indicates an increase in the monthly rate of hospitalisation and an IRR less than 1 is a reduction of the hospitalisation rate. To provide an approximation of the step change of each outcome immediately following reform, we calculated the ratio of the predicted outcomes from January, 2015, over those from December, 2014, as percentages. Recommendations from Turner and colleagues^20^ were used for graphing the IRRs, counterfactuals, and seasonality patterns. All analyses were conducted in Stata (version 16.0) with statistical significance defined as a two-tailed p<0·05.

### Sensitivity analyses

Complete registration of hospital data was mandatory for all Dutch hospitals as of Jan 1, 2013, and therefore data are considered stable over time with no missing data. Before 2013, full registration of hospital data was not mandatory; however, most hospitals participated. Missing data could be a specific hospital, specific treatment, underreporting in a specific month, or random (the number of missing records varies between 1% and 23% within specific months).^21^ To ensure the missing data did not unduly influence our results we performed a sensitivity analysis addressing the effect of the missing hospital data from 2009 to 2012. We did this analysis by comparing the outcomes of the fully adjusted models based on all study data (2009–18) to those based on the data from years with no missing data (2013–18).^21^ Furthermore, we used the Supremum Wald test with symmetric trimming of 15% to identify whether the coefficients in the time-series regression vary over the periods defined by an unknown break date other than our pre-specified break of Jan 1, 2015: the start of the Dutch LTC reform. Additionally, we tested whether the coefficients in the time series varied over the study period defined by other health-care reforms implemented (ie, abolishment of residential care for people with low care needs in 2013 and Outline Agreement Medical Specialist Care 2018)^22,23^ as known break dates. Lastly, the health-care system and people with LTC needs could have adjusted to the Dutch 2015 LTC reform policy changes after a period of time. To test this question, we performed a lagged time series model, omitting the data of the year 2015 (first year post-reform), as sensitivity analysis to detect any potential effect of the maturing of service following reform.

### Role of the funding source

The funders of the study had no role in study design, data collection, data analysis, data interpretation, or writing of the report.

## Results

The monthly hospitalisation rate per 10 000 older adults was significantly higher post-reform (mean 73·07 [SD 5·24]) than pre-reform (mean 63·89 [6·72]), whereas the monthly average LOS was lower post-reform (mean 7·50 days [0·21]) than pre-reform (mean 8·05 days [0·51]; table 1). An overview of unadjusted yearly hospitalisation data is shown in table 2.

**Figure 1:**
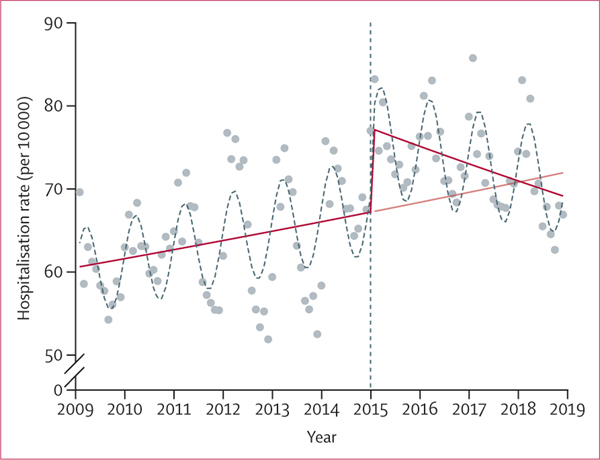
Monthly rates of acute hospitalisation per 10 000 Dutch adults aged 65 years and older, January, 2009–December, 2018 (N=3 777 096) The blue dots show the observed rate of acute monthly hospital admission—eg, in June 2009 there were 58·41 hospitalisations per 10 000 older adults. The red solid line shows the rate of acute monthly hospital admission estimated by the complete reform model (negative binomial regression modelling)—eg, in June 2009 the complete reform model estimated that there were 61·11 hospitalisations per 10 000 older adults. The red dashed line shows the counterfactual that estimated the rate of acute monthly hospital admission if no reform had occurred (negative binomial regression modelling)—eg, in June 2018 the counterfactual estimated that there were 71·43 hospitalisations per 10 000 older adults. However, the complete reform model estimated that in June 2018 there were 69·99 hospitalisations per 10 000 older adults (red solid line). This finding suggests that the rate of monthly hospitalisations estimated by the complete reform model in the post-reform period was lower than that estimated by the counterfactual.

Before the 2015 LTC reform, the rate of acute monthly hospitalisation was increasing: IRR of 1·002 (95% CI 1·001–1·002, p<0·0001; figure 1; table 3). This finding is equivalent to an increase of 0·2% per month (eg, in June, 2009, the model estimated that there were 61·11 hospitalisations per 10 000 older adults, which over a year’s time increased to 62·12 hospitalisations per 10 000 older adults in June, 2010). Immediately after implementation of the reform, there was a positive step change of approximately 11% in the rate of acute monthly hospitalisation (this step change is the vertical rise at the year 2015 in figure 1). This increase was followed by a negative change in trend with an IRR of 0·997 (95% CI 0·996–0·998, p<0·0001), that resulted in a decreasing trend in the post-reform period: IRR of 0·998 (95% CI 0·998–0·999, p=0·0031), which is equivalent to a decrease of 0·2% in the hospitalisation rate per month. Nonetheless, due to the positive step change immediately following reform, the average monthly rate of acute hospitalisation was higher after the reform: IRR of 1·116 (95% CI 1·070–1·165, p<0·0001). This increase is equivalent to the ratio of the least squares means point estimate from the post-reform period (71·66 monthly hospitalisations per 10 000 older adults) over that from the pre-reform period (64·46 monthly hospitalisations per 10 000 older adults). Furthermore, due to the decreasing trend in the post-reform period, in January, 2018, the rate of monthly hospitalisations estimated by the model (70·82 hospitalisations per 10 000 older adults) decreased below the rate of monthly hospitalisations estimated by the counterfactual (70·91 hospitalisations per 10 000 older adults; figure 1). The rate of monthly acute hospitalisation returned to its pre-reform level approximately 4 years after the reform.

**Figure 2:**
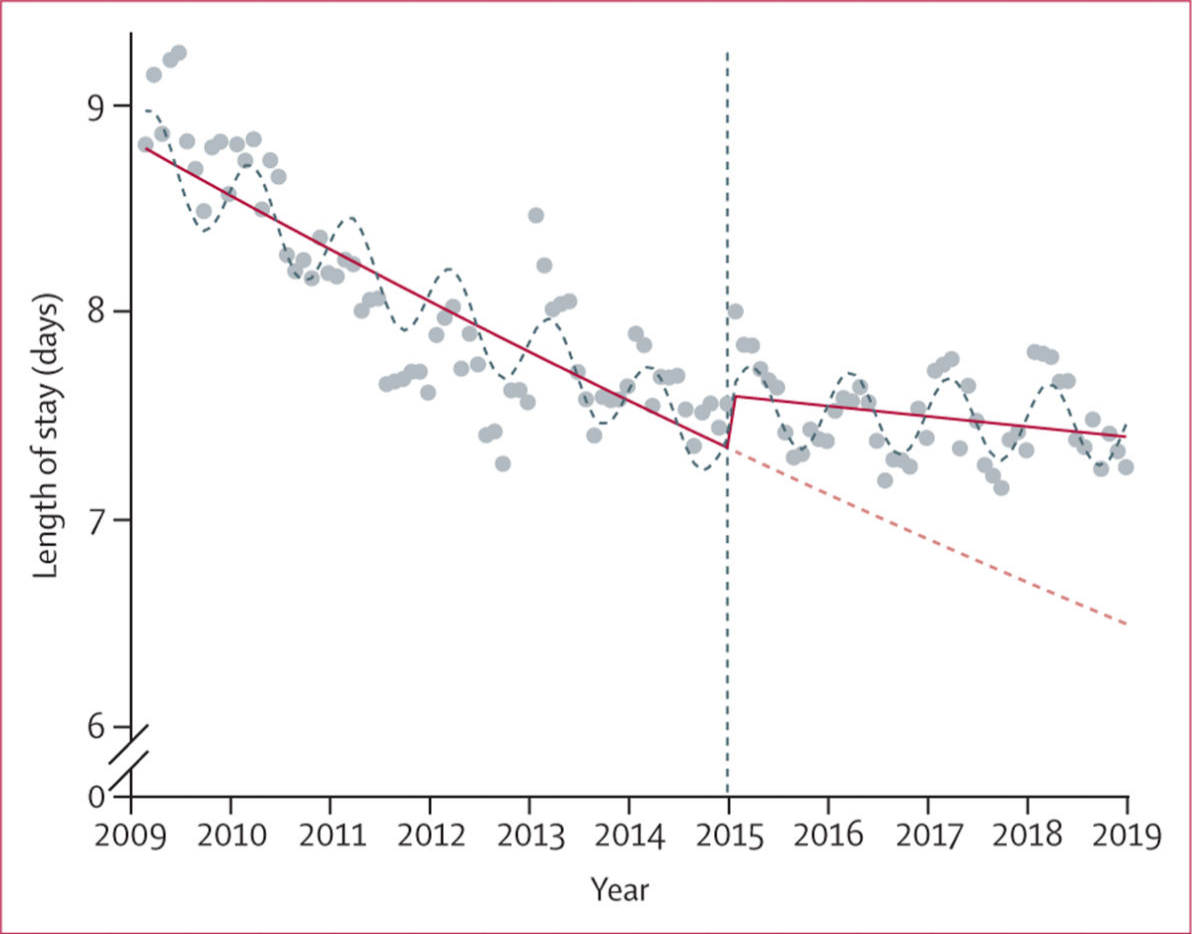
Monthly average LOS, January, 2009–December, 2018 (N=3 777 096) The blue dots show the observed LOS—eg, in June 2009, the observed LOS was 9·26 days. The red solid line shows the LOS estimated by the complete reform model (negative binomial regression modelling)—eg, in June 2009 the complete reform model estimated that the LOS was 8·68 days. The red dashed line shows the counterfactual that estimated LOS if no reform had occurred (negative binomial regression modelling)—eg, in June, 2018, the counterfactual estimated that the LOS was 6·60 days. However, the complete reform model estimated that in June 2018 the LOS was 7·43 days (red solid line). This finding suggests that the LOS estimated by the complete reform model in the post-reform period is greater than that estimated by the counterfactual. LOS=length of stay.

Before the 2015 LTC reform, monthly average LOS of acute hospitalisations was decreasing: IRR of 0·998 (95% CI 0·997–0·998, p<0·0001; figure 2; table 3). This finding is equivalent to a decrease of 0·2% per month (eg, in June, 2009, the model estimated that the monthly average LOS was 8·68 days, which over a year’s time decreased to a monthly average LOS of 8·44 days in June, 2010). Immediately after implementation of the reform, there was a positive step change of approximately 3% in the monthly average LOS (this step change is the vertical rise at the year 2015 in figure 2). This increase was followed by a positive change in trend with an IRR of 1·002 (95% CI 1·002–1·003, p<0·0001) that offset the negative trend exhibited during the pre-reform period, explaining why there was no significant monthly trend in monthly average LOS during the post-reform period (IRR 0·999 [95% CI 0·999–1·000], p=0·11)—ie, no monthly change in LOS. The reform is associated with a negative average change in monthly average LOS during the study period (IRR 0·976 [95% CI 0·960–0·991], p=0·0036), which is equivalent to the ratio of the least squares means point estimate of post-reform (7·74 days) over that of pre-reform (7·93 days). Furthermore, in the post-reform period the monthly average LOS estimated by the model (eg, 7·43 days in June, 2018) is longer compared with the monthly average LOS estimated by the counterfactual (eg, 6·60 days in June, 2018; figure 2).

Sensitivity analyses addressing missing hospitalisation data in the years from 2009 to 2012 showed no meaningful differences in outcomes (appendix 2 p 3). The structural break test for an unknown break date identified the 2015 reform implementation as the single most significant structural break with the highest Wald statistic in both the hospitalisation rate and LOS time series (appendix 2 p 3). The pre-specified break date tests for 2013 (abolishment of residential care for people with low care needs)^22^ detected a significant break in LOS but not in hospitalisation rate (appendix 2 p 3). The pre-specified break date tests for 2018 (the outline Agreement Medical Specialist Care of 2018)^23^ detected a structural break in hospitalisation rate and LOS (appendix 2 p 4). Fully adjusted models setting January, 2013, and January, 2018, as intervention points are presented in appendix 2 (p 4). The sensitivity analysis omitting 2015 data as lagged time series showed strong overlap between the CIs of the estimated coefficients with those from the complete follow-up data. This finding implies there were no meaningful differences in these associations (appendix 2 p 4), which in turn suggests no meaningful effect from the maturation of services.

## Discussion

To our knowledge, this is the first study to report that the 2015 Dutch LTC reform was associated with positive step changes in monthly rates of acute hospitalisation and monthly average LOS among older Dutch adults. Although these findings are consistent with our hypothesis, the findings over the post-reform period suggest that the increase was temporary. In contrast to our hypothesis, the trend in the rate of acute hospitalisations during the post-reform period was negative, resulting in a return to its pre-reform level after approximately 4 years. Despite the decreasing trend in this rate after the reform, the least square means point estimate from the post-reform period of the hospitalisation rate (71·66 monthly hospitalisations per 10 000 older adults) was significantly higher than that from the pre-reform period (64·46 monthly hospitalisation per 10 000 older adults). This finding can be explained by the positive step change immediately following reform. Findings regarding monthly average LOS showed a decreasing pre-reform trend that was cancelled out by the positive change in trend due to the reform, resulting in a stable trend in the post-reform period. This finding explains why the least square means point estimate of LOS from the post-reform period (7·74 hospital days) was significantly lower than pre-reform (7·93 hospital days).

Consistent with our study findings, a Dutch observational study showed that acute hospitalisations of people aged 65–88 years were increasing before the reform implementation in 2015, reporting a 4·8% increase from 1995 to 2009.^9^ Additionally, the study found that the decline in health status of older adults only partially explained the increase in acute hospitalisations, and pointed out that health-care reforms are likely to have contributed.^9^ Also consistent with our findings, publicly available yearly hospital data including the total Dutch population aged 65 years or older showed an increase in the number of clinical hospitalisations pre-reform (2013–14) and a decrease post-reform (2015–19); however, these data included both acute and non-acute hospitalisations.^24^ Our study extends these previous findings by using population-level hospitalisation data over the years 2009–18. In addition, we adjusted our hospitalisation trend for population ageing, which should provide more accurate results. Consistent with our study findings, publicly available hospital data showed a decreasing trend in LOS in the pre-reform period and a stabilisation post-reform.^21^ However, our study specifically studied LOS of acute clinical hospitalisations, which might provide a better-quality indicator for the evaluation of an LTC reform that caused more adults to live at home for as long as possible. In addition, our study used an interrupted time series design using monthly hospital data that enabled us to evaluate if the Dutch 2015 LTC reform was associated with trend changes in acute hospitalisations rates and LOS. To our knowledge this is the first study that has evaluated the association between the Dutch 2015 LTC reform and hospital use and contributes to the understanding of how hospitalisation rates have changed over time in the Netherlands.

This study has several limitations. First, although this study uses monthly population data over a 10-year period, there were small amounts of missing data in the years 2009–12. However, a sensitivity analysis addressing missing data showed no meaningful difference in outcomes. Second, although interrupted time series analyses are usually robust against confounding variables that change slowly over time,^14^ other sociodemographic, health factors, or factors related to the organisation of hospital care (eg, technology and a shift to outpatient and day-care admissions) might have been associated with hospitalisations but not included in the analysis. Moreover, LOS can be influenced by many factors such as mortality, which was not included in the analysis. Third, including a control group in our analysis could have provided stronger evidence to support the relationship between the Dutch 2015 LTC reform and our outcomes. However, there was no correct control group available. Lastly, it is possible that the observed changes could be related to other reforms implemented over the study period. Following a literature review, we found a national LTC reform in 2013 that abolished residential care for people with lower support needs,^22^ and the 2018 Outline Agreement Medical Specialist, a national policy change in hospital care that intended to reduce the annual growth in specialist hospital cost to 0% within 4 years.^23^ The latter was introduced in the final year of our study period minimising the overlap in potential effect. Furthermore, the Wald statistics of the pre-specified break tests of 2013 and 2018 were notably lower than that of the 2015 Dutch LTC reform implementation, which suggests that the magnitude of the break in 2015 was greater than that of the other reforms.

The Dutch 2015 LTC reform included several policies that might explain the increase in the rate of acute hospitalisation. Long-term care-facility admissions were restricted to older adults who required 24-h supervision and care. This restriction would increase the proportion of older adults with frailty living in the community, possibly resulting in a greater risk of acute hospitalisation because their environments at home might not be adapted to their needs. This idea is supported by a Dutch study that showed older adults who were considered eligible for long-term care-facility admission had a reduced probability of having at least one hospital admission.^25^ Furthermore, the responsibility of homecare services was reallocated from the regional single-payers to municipalities and health insurers in addition to home-care budget cuts.^2^ In the first years after the LTC reform, municipalities expressed great difficulties accommodating to their new responsibilities.^3,5^ Therefore, there was limited capacity for innovation and improvements in quality of care that aim to prevent hospitalisations.^5,26^

Following the temporary increase, this study reports a decreasing post-reform trend in hospitalisation rate that is equivalent to a monthly decrease of 0·2%. For example, this meant that over a year period the rate of acute hospitalisation decreased from 72·0 hospitalisations for every 10 000 older adults in June, 2017, to 69·6 hospitalisations for every 10 000 older adults in June, 2018. This finding suggests that the health-care system has gradually adapted to a greater proportion of older adults living in the community. For example, municipalities indicated that a few years after the LTC reform there was more attention for quality of community-based care and support to prevent hospitalisation.^5^ In addition, there was an increase in short-term residential care facilities to bridge the gap between hospital and home. This type of care was designed to prevent hospitalisations of community-residing adults who are not eligible for permanent LTC facility placement and whose health issues temporarily preclude them from living at home for short periods.^27^

Both the immediate increase in average LOS in 2015 and the positive change in trend that mitigated the negative pre-reform period trend, suggest that hospital discharges in older adults might have been delayed after the reform. This delay in discharges might be explained by either an increase in the average severity of the health conditions of the hospitalised older adults or the stricter criteria for LTC facility eligibility. Older adults who before the LTC reform would have been discharged from hospital to an LTC facility are now kept in the hospital longer to recover to return home or wait for LTC facility placement. In 2019, 1% of the hospital beds were occupied by individuals without a medical need to stay in hospital, but who were eligible and waiting for LTC facility placement.^28^ The idea that LTC facility entry decreases length of hospital stay in the older adult population is supported by a Portuguese study.^29^ It is estimated that the Netherlands needs 123 000 extra LTC facility beds by 2040 to keep up with the demand of the growing older adult population.^30^ This estimation, in combination with shortage of LTC facility staff,^31^ might increase hospital LOS and delays in discharge in the future. It is crucial to better understand how policies in long-term care affect outcomes in curative care.

This study provides evidence that the 2015 Dutch LTC reform was associated with a direct increase in the monthly rate of acute hospitalisations. This increase was followed by a decreasing trend that returned the hospitalisation rate to its pre-reform level in approximately 4 years, suggesting that the health-care system slowly adapted to frailer adults living in the community. The pre-reform decreasing trend in LOS was eliminated by the reform implementation, which might indicate that hospital discharges of older adults are being delayed or that older adults enter the hospital with more severe health problems requiring longer care. The challenge that policy makers in many countries, including the Netherlands, must deal with is how to best provide accessible and good quality care for their ageing populations at a fiscally sustainable price. To divide optimal policies, policy makers must trade off health and broader wellbeing effects to costs of different care options. Previous research has evaluated effects of LTC reforms from various countries on dimensions such as home-care use,^32^ wellbeing,^33^ and location of death.^11,34^ In this Article, we have considered another important dimension, but just one of the many that are relevant. Future research should include other clinical, person-centred, social, and economic outcomes to arrive at a more complete assessment of the welfare effects of Dutch 2015 LTC reform.

## Supplementary Material

Supplementary Appendix 1 in Dutch

Supplementary Appendix 2 in English

## Figures and Tables

**Table 1:** Study cohort characteristics, 2009–18

Patient-level characteristics	Pre-reform (N= 2 054 018)	Post-reform (N= 1723078)	p value

Age, years	78 (71–84)	78 (71–84)	<0·0001*
Sex	∙∙	∙∙	<0·0001†
Female	1 043 692 (50·81%)	851 540 (49·42%)	∙∙
Male	1 010 326 (49·19%)	871 538 (50·58%)	∙∙
**Hospital-level characteristics**			
Monthly number of hospitalisations	28 639 (3509)	35 883 (2351)	<0·0001*
;Monthly hospitalisation rate‡	63·89 (6·72)	73·07 (5·24)	<0·0001*
Monthly length of stay in days	8·05 (0·51)	7·50 (0·21)	<0·0001*

Data are median (IQR), n (%), or mean (SD).

* Independent *t*-test.

† χ^2^ test.

‡ Monthly hospitalisation rate=(monthly hospitalisations/monthly standardised total population aged 65 years and older) × 10 000.

**Table 2:** Unadjusted yearly hospital use data, 2009–18 (N=3 777 096 hospitalisations)

	Mean monthly hospitalisations	Mean monthly hospitalisation rate*	Mean length of stay in days

2009	25 455 (1650)	59·87 (4·04)	8·87 (0·25)
2010	27 245 (1146)	63·11 (2·75)	8·48 (0·26)
2011	27 553 (2423)	62·59 (6·00)	7·91 (0·25)
2012	29 090 (4169)	64·31 (9·68)	7·69 (0·24)
2013	29 366 (3381)	63·43 (7·70)	7·83 (0·33)
2014	33 123 (1895)	70·00 (4·11)	7·61 (0·16)
2015†	35 893 (1747)	74·70 (3·84)	7·58 (0·24)
2016†	36 329 (2210)	74·49 (4·72)	7·44 (0·15)
2017†	35 879 (2417)	72·49 (5·12)	7·46 (0·22)
2018†	35 390 (2123)	70·38 (6·53)	7·52 (0·22)

Data are mean (SD).

* Monthly hospitalisation rate per 10 000 adults aged 65 years and older.

† Post-reform years.

**Table 3:** Adjusted IRR of acute clinical hospitalisation rate and average LOS from interrupted time series analysis (N=3 777 096 hospitalisations)

	Hospitalisation rate IRR (95% CI); p value	Average LOS IRR (95% CI); p value

Pre-reform trend	1·002 (1·001–1·002); p<0·0001	0·998 (0·997–0·998); p<0·0001
Trend change (interaction of pre-reform trend and reform)	0·997 (0·996–0·998); p<0·0001	1·002 (1·002–1·003); p<0·0001
Post-intervention trend (linear combination of pre-reform trend and trend change)	0·998 (0·998–0·999); p=0·0031	0·999 (0·999–1·000); p=0·11
Average reform effect	1·116 (1·070–1·165); p<0·0001	0·976 (0·960–0·991); p=0·0036

IRR=incidence rate ratios. LOS=length of stay.

## Research in context

### Evidence before this study

We searched the Scopus database for articles published between Jan 31, 2010, and Jan 31, 2022, using the search terms “long-term care” AND “reform” OR “policy” AND “hospital*” OR “hospital utilization” with no language restrictions. Of the 322 studies identified, only two Spanish studies found that long-term care (LTC) reforms that increased subsidising of community-based LTC services were associated with decreased hospital admission. Our own previous work showed that LTC reforms from the Netherlands, Japan, and Norway were associated with shifts in proportions of deaths occurring in hospital, suggesting potential associations with changes in the use of hospital care. To our knowledge, no previous studies have investigated the effect of a national LTC reform on acute hospital use by the use of interrupted time series analyses.

### Added value of this study

This is the first study to report that the 2015 Dutch LTC reform was associated with positive step changes in monthly rates of acute hospitalisation and monthly average length of stay (LOS) among older Dutch adults. Our findings suggest that the increase in the rate of acute hospitalisation after the reform implementation was temporary, whereas the increase in LOS post-reform appeared to last longer than expected. These results have the potential to inform policy makers about effects of ageing-in-place LTC strategies on health and curative care.

### Implications of all the available evidence

This study corroborates existing evidence from other countries informing that LTC reforms can result in changes in hospital use and extends these findings by using population data describing longitudinal trends. Future research should include other clinical, person-centred, social, and economic outcomes to holistically assess policy changes.
