# Supplementary material for: Acute hospital use in older adults following the 2015 Dutch reform of long-term care: an interrupted time series analysis
Source: Lancet Healthy Longev. Author manuscript; Available in PMC 2023 Jul 3. (PMC10316520; doi:10.1016/S2666-7568(23)00064-8)
Supplement: Supplementary Appendix 1 in Dutch [file NIHMS1905960-supplement-Supplementary_Appendix_1_in_Dutch.pdf]

THE LANCET
Healthy Longevity

Supplementary appendix 1

This translation in Dutch was submitted by the authors and we reproduce it as supplied. It has not been peer reviewed. *The Lancet*'s editorial processes have only been applied to the original in English, which should serve as reference for this manuscript.

Deze vertaling in het Nederlands werd door de auteurs toegevoegd en we reproduceren de tekst zoals deze werd aangeleverd. Dit Nederlandstalig abstract onderging geen peer review. Het redactioneel proces van The Lancet werd enkel uitgevoerd op de originele Engelse tekst, welke geldt als de referentie voor dit artikel.

Supplement to: Wammes JD, Bakx P, Wouterse B, Buurman BM, Murphy TE, MacNeil Vroomen JL. Acute hospital use in older adults following the 2015 Dutch reform of long-term care: an interrupted time series analysis. *Lancet Healthy Longev* 2023; **4:** e257–64.

Samenvatting

Achtergrond De Nederlandse regering implementeerde op 1 januari 2015 de hervorming langdurige zorg (HLZ), die onder andere gericht was op ouderen zo lang mogelijk thuis te laten wonen. Een toename van het aantal thuiswonende ouderen kan mogelijk hebben geleid tot meer acute ziekenhuisopnames. Het doel van deze studie was te evalueren of de HLZ geassocieerd was met een toename in het aantal maandelijkse acute ziekenhuisopnames per 10.000 ouderen (≥65 jaar), en of de HLZ geassocieerd was met een toename in de gemiddelde ligduur in dagen.

Methode We gebruikten nationale ziekenhuisdata (verstrekt door Dutch Hospital Data) van de jaren 2009-2018 op individueel opname niveau. Alle acute klinische ziekenhuisopnames waarvoor een medisch specialist besloot dat behandeling binnen 24 uur noodzakelijk was, werden geïncludeerd. We analyseerden met gebruik van een 'interrupted time series design' de associatie tussen de HLZ en het aantal acute opnames, en de associatie tussen de HLZ en de gemiddelde ligduur. De analyse werd gecontroleerd voor bevolkingsgroei (bevolkingsregister data werd verstrekt door het CBS) en seizoensgebonden trends. Resultaten werden gepresenteerd als 'incident rate ratios' (IRR).

Resultaten In de jaren voor de introductie van de HLZ was de trend in het aantal acute ziekenhuisopnames per 10.000 ouderen aan het toenemen met 0.2% per maand (IRR 1·002 [95% CI 1·001-1·002]). De introductie van de HLZ ging gepaard met een toename in het aantal opnames waardoor een positief gemiddeld hervormingseffect werd waargenomen (1·116 [1·070-1·165]). In de jaren na de HLZ was juist een dalende trend te zien van 0.2% per maand (0·998 [0·998-0·999]), waardoor in 2019 het aantal opnames weer bijna gelijk was aan de situatie net voor de introductie van de HLZ. De gemiddelde ligduur in dagen liet voor de introductie van de HLZ een dalende trend zien van 0.2% per maand (0·998 [0·997-0·998]). De introductie van de HLZ resulteerde in een stabilisatie van de trend in de periode na de hervorming (0·999 [0·999-1·000]), wat ervoor zorgde dat de gemiddelde ligduur in de periode na de HLZ bij benadering gelijk bleef.

Interpretatie Deze resultaten wijzen erop dat de stijging van het aantal acute ziekenhuisopnames na de HLZ tijdelijk was, terwijl de dalende trend in de ligduur na de HLZ zich niet heeft voortgezet. Deze bevindingen kunnen beleidsmakers informeren over de effecten van het langer thuis wonen van ouderen op de curatieve gezondheidszorg.

Financiering ZonMw, the Yale Claude Pepper Center en the National Center for Advancing Translational Sciences, National Institutes of Health.
