# Supplementary material for: Acute hospital use in older adults following the 2015 Dutch reform of long-term care: an interrupted time series analysis
Source: Lancet Healthy Longev. Author manuscript; Available in PMC 2023 Jul 3. (PMC10316520; doi:10.1016/S2666-7568(23)00064-8)
Supplement: Supplementary Appendix 2 in English [file NIHMS1905960-supplement-Supplementary_Appendix_2_in_English.pdf]

THE LANCET
Healthy Longevity

Supplementary appendix 2

This appendix formed part of the original submission and has been peer reviewed. We post it as supplied by the authors.

Supplement to: Wammes JD, Bakx P, Wouterse B, Buurman BM, Murphy TE, MacNeil Vroomen JL. Acute hospital use in older adults following the 2015 Dutch reform of long-term care: an interrupted time series analysis. *Lancet Healthy Longev* 2023; **4:** e257–64.

Appendix

STROBE guidelines

	Item No	Recommendation	
**Title and abstract**	1	(*a*) Indicate the study's design with a commonly used term in the title or the abstract	√
		(*b*) Provide in the abstract an informative and balanced summary of what was done and what was found	√
**Introduction**			
Background/rationale	2	Explain the scientific background and rationale for the investigation being reported	√
Objectives	3	State specific objectives, including any prespecified hypotheses	√
**Methods**			
Study design	4	Present key elements of study design early in the paper	√
Setting	5	Describe the setting, locations, and relevant dates, including periods of recruitment, exposure, follow-up, and data collection	√
Participants	6	(*a*) Give the eligibility criteria, and the sources and methods of selection of participants. Describe methods of follow-up	√
		(*b*) For matched studies, give matching criteria and number of exposed and unexposed	
Variables	7	Clearly define all outcomes, exposures, predictors, potential confounders, and effect modifiers. Give diagnostic criteria, if applicable	√
Data sources/ measurement	8	For each variable of interest, give sources of data and details of methods of assessment (measurement). Describe comparability of assessment methods if there is more than one group	√
Bias	9	Describe any efforts to address potential sources of bias	√
Study size	10	Explain how the study size was arrived at	√
Quantitative variables	11	Explain how quantitative variables were handled in the analyses. If applicable, describe which groupings were chosen and why	√
Statistical methods	12	(*a*) Describe all statistical methods, including those used to control for confounding	√
		(*b*) Describe any methods used to examine subgroups and interactions	N/A
		(*c*) Explain how missing data were addressed	√
		(*d*) If applicable, explain how loss to follow-up was addressed	N/A
		(*e*) Describe any sensitivity analyses	√
**Results**			
Participants	13*	(a) Report numbers of individuals at each stage of study—eg numbers potentially eligible, examined for eligibility, confirmed eligible, included in the study, completing follow-up, and analysed	√
		(b) Give reasons for non-participation at each stage	N/A
		(c) Consider use of a flow diagram	N/A
Descriptive data	14*	(a) Give characteristics of study participants (eg demographic, clinical, social) and information on exposures and potential confounders	√
		(b) Indicate number of participants with missing data for each variable of interest	N/A
		(c) Summarise follow-up time (eg, average and total amount)	N/A
Outcome data	15*	Report numbers of outcome events or summary measures over time	√

Main results	16	(*a*) Give unadjusted estimates and, if applicable, confounder-adjusted estimates and their precision (eg, 95% confidence interval). Make clear which confounders were adjusted for and why they were included<br>(*b*) Report category boundaries when continuous variables were categorized<br>(*c*) If relevant, consider translating estimates of relative risk into absolute risk for a meaningful time period	√<br>N/A<br>N/A
Other analyses	17	Report other analyses done—eg analyses of subgroups and interactions, and sensitivity analyses	√
**Discussion**			
Key results	18	Summarise key results with reference to study objectives	√
Limitations	19	Discuss limitations of the study, taking into account sources of potential bias or imprecision. Discuss both direction and magnitude of any potential bias	√
Interpretation	20	Give a cautious overall interpretation of results considering objectives, limitations, multiplicity of analyses, results from similar studies, and other relevant evidence	√
Generalisability	21	Discuss the generalisability (external validity) of the study results	√
**Other information**			
Funding	22	Give the source of funding and the role of the funders for the present study and, if applicable, for the original study on which the present article is based	√

Table 1. Model selection process based on minimization of the BIC for average length of stay (LOS).[1]

Model outcome	Distribution	BIC
LOS Mean	Negative binomial	-559.97
LOS Median	Negative binomial	-545.35
LOS Mean	Gaussian	-535.13
LOS Median	Gaussian	-530.78

Interrupted time series model details

Technical Details of the Interrupted Time Series Models

"We regressed the outcomes (monthly rate of hospitalization and monthly average length of stay) in two separate negative binomial models that each regressed on continuous time in months (T), an indicator of reform (X_1), and their interaction (TX_1). We used the following interrupted time series model approach as described by Bernal et al.[2]

$$\log Y_t = \beta_0 + \beta_1 T + \beta_2 X_1 + \beta_3 TX_1$$

where β_0 represents the outcome at the start of data collection (January 2009). β_1 is interpreted as the change in the log of the outcome associated with incremental rise in months of time (T) and represents the trend during the pre-reform period. β_2 is the coefficient of the indicator of reform (X_1: pre-reform coded as 0 and post-reform coded as 1) and represents the average change in the log of the outcome associated with the reform (i.e. average reform effect). β_3 is the coefficient of the interaction term between Time and Reform, and represents the change in trend in the post-reform period relative to that of the pre-reform period. In addition, the post-reform trend was calculated by linear combination of the β_1 and β_3 coefficients and is interpreted as the change in the log of the outcome associated with months of Time in the post-reform period. Because the model is fit on the log of the outcome, all results are reported as incident rate ratios (IRR), which are the exponentiation of the regression coefficients and their corresponding 95% confidence intervals."

Table 2. Adjusted incident rate ratios (IRR)* from sensitivity analysis using only years with complete data (2013-2018).

Interrupted time series parameters	Hospitalization rate<br>β, IRR (95% CI) *P*-value	Length of stay<br>β, IRR (95% CI) *P*-value
Pre-reform trend	0.005, 1.005 (1.001-1.008) 0.004	-0.002, 0.998 (0.996-0.999) < 0.001
Slope change	-0.006, 0.994 (0.990-0.997) < 0.001	0.002, 1.002 (1.001-1.003) < 0.001
Average reform effect	0.075, 1.078 (1.033-1.126) 0.008	-0.006, 0.994 (0.976-1.011) 0.467

*IRR is the exponentiation of the regression parameter with a value > 1 indicating an increase and a value of <1 indicating a decrease.

Table 3. Structural break test for unknown break date* detected January 2015, the Dutch Long-term care reform implementation month, as break date with highest Wald statistic.

Interrupted time series parameters	Wald statistic	*P* - value
Hospitalization rate	50.279	< 0.001
Length of stay	165.243	< 0.001

*Tests whether the coefficients in a time-series regression vary over the periods defined by an unknown break date.[3]

Table 4. Interrupted Time Series Structural break test for known break date*, elimination of residential care for people with low care needs (January 2013)[4]**.**

Interrupted time series parameters	Wald statistic	*P* - value
Hospitalization rate	6.252	0.044
Length of stay	113.104	< 0.001

*Test of whether the coefficients in a time-series regression vary over the periods defined by known break dates.[5]

Table 5. Interrupted Time Series Structural break test for known break date*, Outline Agreement Medical Specialist Care (January 2018).[6]

Interrupted time series parameters	Wald statistic	*P* - value
Hospitalization rate	17.199	< 0.001
Length of stay	27.836	< 0.001

*Test of whether the coefficients in a time-series regression vary over the periods defined by known break dates.[5]

Table 6. Adjusted incident rate ratios (IRR)* with reform dummy and interaction set at January 2013 (elimination of residential care for people with low care needs).[4]

Interrupted time series parameters	Hospitalization rate IRR (95% CI) *P*-value	Length of stay IRR (95% CI) *P*-value
Pre-reform trend	1.001 (0.999-1.002) 0.105	0.996 (0.995-0.996) < 0.001
Slope change	1.000 (0.999-1.002) 0.661	1.003 (1.003-1.004) < 0.001
Average reform effect	1.046 (0.994-1.100) 0.084	0.959 (0.945-0.974) < 0.001

*IRR is the exponentiation of the regression parameter with a value > 1 indicating an increase and a value of <1 indicating a decrease.

Table 7. Adjusted incident rate ratios (IRR)* with reform dummy and interaction set at January 2018 (Outline Agreement Medical Specialist Care).[6]

Interrupted time series parameters	Hospitalization rate IRR (95% CI) *P*-value	Length of stay IRR (95% CI) *P*-value
Pre-reform trend	1.002 (1.002-1.003) < 0.001	0.998 (0.998-0.999) < 0.001
Slope change	0.994 (0.982-1.007) 0.362	0.998 (0.993-1.003) 0.484
Average reform effect	0.947 (0.867-1.034) 0.227	0.936 (0.900-0.973) 0.001

*IRR is the exponentiation of the regression parameter with a value > 1 indicating an increase and a value of <1 indicating a decrease.

Table 8. Adjusted incident rate ratios (IRR)* from sensitivity analysis lagged time series, omitting 2015 data from the analysis.

Interrupted time series parameters	Hospitalization rate IRR (95% CI) *P*-value	Length of stay IRR (95% CI) *P*-value
Pre-reform trend	1.002 (1.001-1.002) < 0.001	0.997 (0.997-0.997) < 0.001
Slope change	0.996 (0.994-0.998) < 0.001	1.003 (1.002-1.004) < 0.001
Average reform effect	1.132 (1.053-1.217) < 0.001	0.969 (0.968-0.970) < 0.001

*IRR is the exponentiation of the regression parameter with a value > 1 indicating an increase and a value of <1 indicating a decrease.

References

1. Raftery AE. Bayesian model selection in social research. *Sociol Methodol* 1995; **25**: 111-63.
2. Bernal JL, Cummins S, Gasparrini A. Interrupted time series regression for the evaluation of public health interventions: a tutorial. *Int J Epidemiol* 2017; **46**: 348-55.
3. Stata. *Test for a structural break with an unknown break date*. [cited October 7, 2022]; Available from: https://www.stata.com/manuals/tsestatsbsingle.pdf
4. Dutch Ministry of Health Welfare and Sport. Extramuraliseren lichte zorgzwaarte-pakketten. 2012.
5. Stata. *Test for a structural break with a known break date*. [cited October 7, 2022]; Available from: https://www.stata.com/manuals/tsestatsbknown.pdf
6. Dutch Ministry of Health Welfare and Sport. Bestuurlijk akkoord medisch-specialistische zorg. 2018.
